# Acute Esophageal Necrosis Syndrome (Black Esophagus): A Case Report of Rare Presentation

**DOI:** 10.7759/cureus.24276

**Published:** 2022-04-19

**Authors:** George Trad, Nazanin Sheikhan, Jason Ma, Abdul Gader Gheriani, Ahmed Sagaslli

**Affiliations:** 1 Internal Medicine, MountainView Hospital, Las Vegas, USA

**Keywords:** clinical gastroenterology, end of life and hospice care, medical intensive care unit (micu), hepatocellular carcinoma (hcc), acute esophageal necrosis (aen)

## Abstract

Acute esophageal necrosis (AEN) is a rare clinical finding due to multifocal factors consisting of an ischemic insult to the esophagus, corrosive injury from gastric content, and diminished mucosal defense. It is also referred to as “black esophagus” or acute necrotizing esophagitis. The clinical presentation mainly consists of upper gastrointestinal bleed and abdominal pain. Associated symptoms include nausea, vomiting, and dysphagia. AEN can be diagnosed by esophagogastroduodenoscopy (EGD) with findings of diffuse circumferential black pigmentation in the distal esophagus that classically extends to the gastroesophageal junction. A diagnostic biopsy is not required but recommended. Treatment of AEN is conservative management to maintain hemodynamic stability and treat coexisting medical conditions. Herein, we present the case of a 78-year-old male who initially presented with hematemesis and abdominal discomfort of five-day duration and was subsequently found to have AEN.

## Introduction

Acute esophageal necrosis (AEN) is a rare clinical finding that can present in patients following an ischemic injury to the esophagus. It is also known as “black esophagus” given its presentation on esophagogastroduodenoscopy and was first described in 1990 by Goldenberg et al [1]. The clinical presentation is mainly upper gastrointestinal bleed with complaints of abdominal discomfort [1]. The treatment approach is targeted toward conservative management by maintaining nothing by mouth (NPO) status, administering IV fluids and IV proton-pump inhibitors, and treating the underlying disease. Complications such as esophageal perforation can occur following the development of AEN, therefore close clinical monitoring is required [2]. In general, the presence of AEN usually indicates a poorer prognosis and higher mortality rate [2].

## Case presentation

A 78-year-old male with a past medical history of adenocarcinoma of the lung, hepatocellular carcinoma, prostate cancer, coronary artery disease, chronic obstructive pulmonary disease, active tobacco use disorder, and alcohol dependence presented to the emergency department after five days of hematemesis. Associated symptoms included shortness of breath and abdominal discomfort. On presentation, vital signs were as follows: body temperature 38.0°C, blood pressure 80/40 mmHg, heart rate 140 beats/min, respiratory rate 20 breaths/min, and oxygen saturation 85% on room air. Physical examination was noted for overall appearance of a chronically ill-appearing man with poor dentition, cardiovascular: irregular heart rate rhythm, abdomen: distended with a large ventral hernia, and extremities: +2 pitting edema on bilateral lower extremities. Initial laboratory studies including complete blood count, chemistry panel, and hepatic function panel were notable for an elevated white blood cell count, blood urea nitrogen (BUN), creatinine level, and total bilirubin (Table 1). Model for End-stage Liver Disease (MELD)-Na score was 17 with an estimated 4% 90-Day mortality rate.

**Table 1:** Initial laboratory values on admission

Laboratory	Value	Reference range
White blood cell (10^3^/µL)	13.5	4.8-10.8
Hemoglobin (gm/dL)	14.8	14.0-18.0
Hematocrit (%)	48.3	42.0-52.0
Platelets (10^3^/µL)	342	150-450
Blood urea nitrogen (mg/dL)	34	7-18
Creatinine (mg/dL)	2.14	0.52-1.23
Aspartate aminotransferase (U/L)	23	15-37
Alanine transaminase (U/L)	16	12-78
Alkaline phosphatase (U/L)	158	45-117
Total bilirubin (mg/dL)	2.1	0.1-1.0

**Figure 1:**
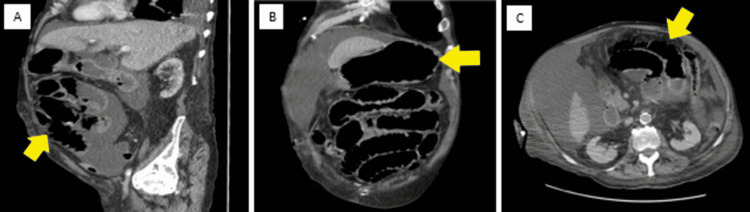
A) sagittal, B) coronal, and C) axial: presence of small bowel obstruction on abdominal/pelvis CT.

Computed tomography (CT) of the abdomen and pelvis with IV contrast is shown in Figure 1.

**Figure 2:**
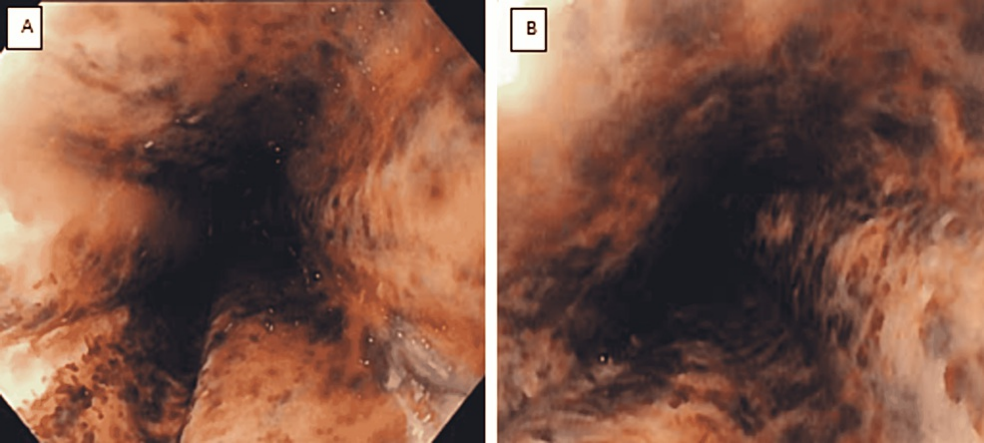
A-B) Endoscopic finding of black esophagus: acute esophageal necrosis.

The scan demonstrated diffuse distention of the small bowel without discrete transition point in the anterior pelvis compatible with small bowel obstruction (SBO). In addition, a large dominant complex and low attenuation right liver mass measuring approximately 9.1 cm anteroposterior (AP), 6.1 cm transverse, and 9.1 cm in height were noted. An echocardiogram noted mild aortic stenosis, mild tricuspid regurgitation, and an ejection fraction of 55%. Fungal cultures were negative. The patient was admitted to the intensive care unit (ICU). The patient had an EGD (Figure 2).

On the day of presentation and the findings consisted of a diffuse severely ulcerated esophagus with exudation, dark pigmentation, and black mucosal surface. The patient remained in the ICU for a total of 5 days. During his stay, he had a nasogastric tube placed with intermittent suction. On the third day of admission, SBO resolved but a swallow evaluation trial was unsuccessful due to the risk of aspiration. Oncological surgery and gastroenterology teams reported a guarded prognosis given the patient’s poor presentation, worsening kidney function, and history of multiple cancers with no prior treatment. On day five of hospitalization, the patient and family elected for hospice care.

## Discussion

AEN syndrome or “black esophagus” is a rare finding following an ischemic insult to the esophagus. It was first reported by Goldenberg et al. in 1990 and labeled as a syndrome by Gurvits et al. in 2007 [1,3,4]. The pathophysiology stems from a combination of hypoperfusion to the esophagus, corrosive injury from gastric contents, and decreased function of mucosal barrier systems [3,5]. Elderly male patients and those with chronic medical conditions such as malignancy, diabetes mellitus, cardiovascular compromise, and malnourishment are at higher risks of developing AEN [6].

The typical clinical presentation consists of upper gastrointestinal bleed manifesting as hematemesis, coffee-ground emesis, and/or melena. Additional symptoms include abdominal pain, vomiting, and dysphagia [3]. AEN can be diagnosed by an EGD and characteristic findings are patchy or circumferential black discolorations with underlying friable hemorrhagic tissue [1]. The development of AEN carries a poor prognosis and the medical approach is mainly targeted at treating coexisting medical diseases [2]. Generally, these patients will need fluid maintenance, IV proton-pump inhibitors, and maintain NPO status. It is still debatable whether to give antibiotics given the inherent risks of antibiotics causing AEN [4]. Currently, there are no reported studies supporting the use of antibiotics in patients with AEN. Esophageal perforation is one of the major complications seen in patients with AEN that requires an emergent surgical intervention [2].

Our patient was an elderly male with a past medical history of multiple malignancies including hepatocellular carcinoma and adenocarcinoma of the lung. He presented to the hospital with reported hematemesis. Initial vitals were notable for low blood pressure which contributed to the tissue hypoperfusion. The patient’s abdominal CT scan reported a questionable SBO and patient-reported multiple vomiting episodes, which together indicated that the patient likely had a corrosive esophageal injury from gastric content. The combination of tissue hypoperfusion, esophageal injury from gastric content, and malnourishment contributed to the development of AEN.

## Conclusions

AEN or “black esophagus” is a rare fatal clinical finding that follows an ischemic esophageal injury. The typical presentation consists of upper gastrointestinal bleed, abdominal pain, and hemodynamic instability. Treatment is directed mainly toward preventing further damage to the esophagus by keeping patients NPO and maintaining hemodynamic stability. Patients with AEN require higher levels of care and need to be monitored closely due to potential complications. These patients usually present with other significant comorbidities that require close monitoring as well. The presence of AEN in patients is associated with a higher mortality rate, which reflects the complicated underlying medical conditions these patients usually present with.
